# Epidemic Cholera

**Published:** 1834

**Authors:** 


### III. Epidemic Cholera.

It appears by the gazettes, that an occasional case of Cholera occurs on board the steam boats, which ply between New-Orleans and Cincinnati.

In this city, about the time of the *equinox*, when a thunder shower was succeeded by great depression of temperature, several cases of diarrhœa, and one or two of fatal Cholera occurred. A week has since elapsed, without any subsequent death, and indeed, there does not at present seem to be any tendency to the Epidemic.

